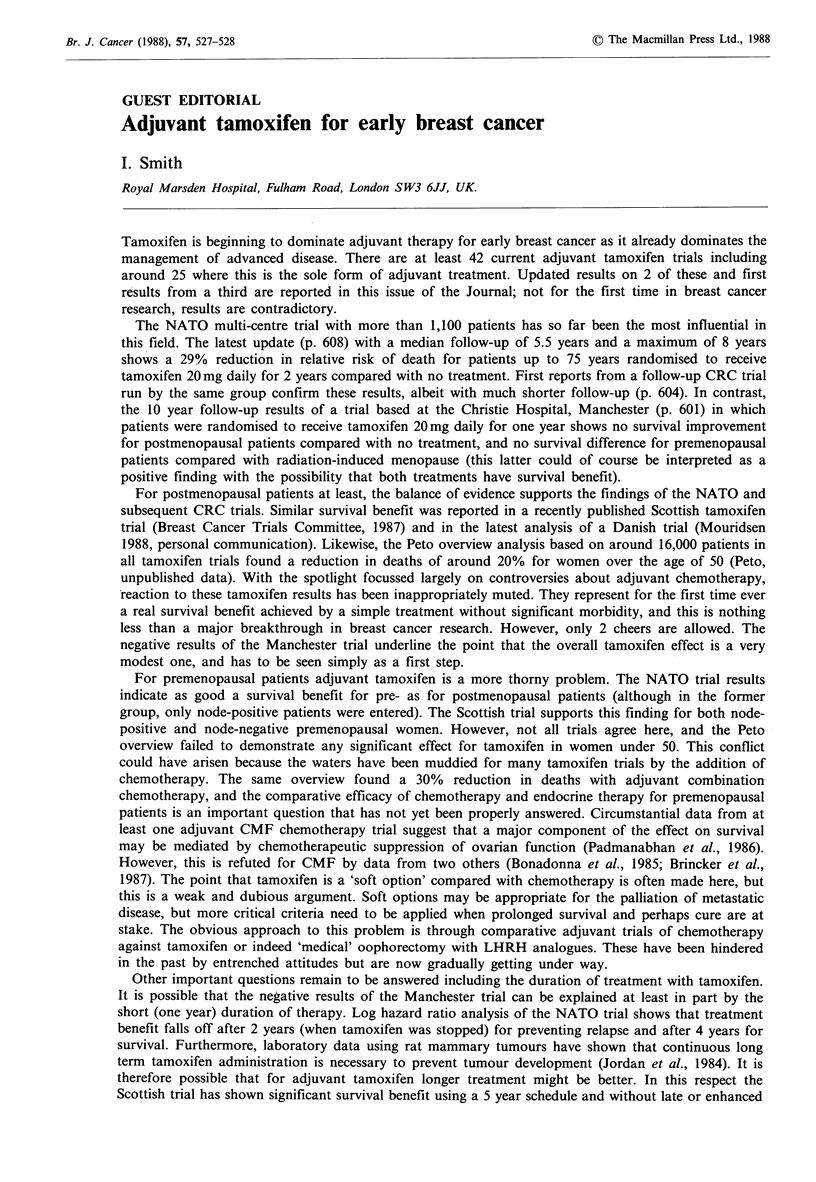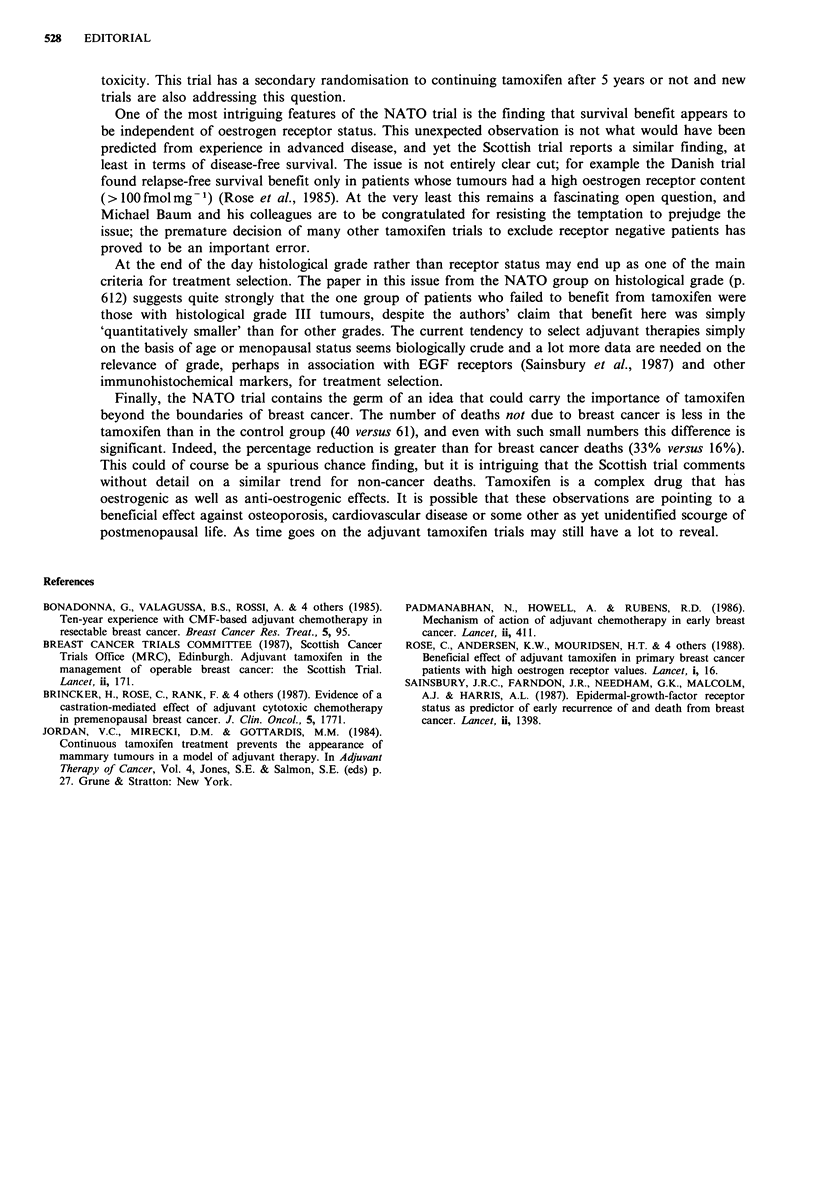# Adjuvant tamoxifen for early breast cancer.

**DOI:** 10.1038/bjc.1988.122

**Published:** 1988-06

**Authors:** I. Smith


Br. J. Cancer (1988), 57, 527-528 © The Macmillan Press Ltd., 1988

GUEST EDITORIAL

### Adjuvant tamoxifen for early breast cancer

I. Smith

*Royal Marsden Hospital, Fulham Road, London SW3 6JJ, UK.*

Tamoxifen is beginning to dominate adjuvant therapy for early breast cancer as it already dominates the management of advanced disease. There are at least 42 current adjuvant tamoxifen trials including around 25 where this is the sole form of adjuvant treatment. Updated results on 2 of these and first results from a third are reported in this issue of the Journal; not for the first time in breast cancer research, results are contradictory.

The NATO multi-centre trial with more than 1,100 patients has so far been the most influential in this field. The latest update (p. 608) with a median follow-up of 5.5 years and a maximum of 8 years shows a 29% reduction in relative risk of death for patients up to 75 years randomised to receive tamoxifen 20 mg daily for 2 years compared with no treatment. First reports from a follow-up CRC trial run by the same group confirm these results, albeit with much shorter follow-up (p. 604). In contrast, the 10 year follow-up results of a trial based at the Christie Hospital, Manchester (p. 601) in which patients were randomised to receive tamoxifen 20 mg daily for one year shows no survival improvement for postmenopausal patients compared with no treatment, and no survival difference for premenopausal patients compared with radiation-induced menopause (this latter could of course be interpreted as a positive finding with the possibility that both treatments have survival benefit).

For postmenopausal patients at least, the balance of evidence supports the findings of the NATO and subsequent CRC trials. Similar survival benefit was reported in a recently published Scottish tamoxifen trial (Breast Cancer Trials Committee, 1987) and in the latest analysis of a Danish trial (Mouridsen 1988, personal communication). Likewise, the Peto overview analysis based on around 16,000 patients in all tamoxifen trials found a reduction in deaths of around 20% for women over the age of 50 (Peto, unpublished data). With the spotlight focussed largely on controversies about adjuvant chemotherapy, reaction to these tamoxifen results has been inappropriately muted. They represent for the first time ever a real survival benefit achieved by a simple treatment without significant morbidity, and this is nothing less than a major breakthrough in breast cancer research. However, only 2 cheers are allowed. The negative results of the Manchester trial underline the point that the overall tamoxifen effect is a very modest one, and has to be seen simply as a first step.

For premenopausal patients adjuvant tamoxifen is a more thorny problem. The NATO trial results indicate as good a survival benefit for pre- as for postmenopausal patients (although in the former group, only node-positive patients were entered). The Scottish trial supports this finding for both node-positive and node-negative premenopausal women. However, not all trials agree here, and the Peto overview failed to demonstrate any significant effect for tamoxifen in women under 50. This conflict could have arisen because the waters have been muddied for many tamoxifen trials by the addition of chemotherapy. The same overview found a 30% reduction in deaths with adjuvant combination chemotherapy, and the comparative efficacy of chemotherapy and endocrine therapy for premenopausal patients is an important question that has not yet been properly answered. Circumstantial data from at least one adjuvant CMF chemotherapy trial suggest that a major component of the effect on survival may be mediated by chemotherapeutic suppression of ovarian function (Padmanabhan *et al.*, 1986). However, this is refuted for CMF by data from two others (Bonadonna *et al.*, 1985; Brincker *et al.*, 1987). The point that tamoxifen is a 'soft option' compared with chemotherapy is often made here, but this is a weak and dubious argument. Soft options may be appropriate for the palliation of metastatic disease, but more critical criteria need to be applied when prolonged survival and perhaps cure are at stake. The obvious approach to this problem is through comparative adjuvant trials of chemotherapy against tamoxifen or indeed 'medical' oophorectomy with LHRH analogues. These have been hindered in the past by entrenched attitudes but are now gradually getting under way.

Other important questions remain to be answered including the duration of treatment with tamoxifen. It is possible that the negative results of the Manchester trial can be explained at least in part by the short (one year) duration of therapy. Log hazard ratio analysis of the NATO trial shows that treatment benefit falls off after 2 years (when tamoxifen was stopped) for preventing relapse and after 4 years for survival. Furthermore, laboratory data using rat mammary tumours have shown that continuous long term tamoxifen administration is necessary to prevent tumour development (Jordan *et al.*, 1984). It is therefore possible that for adjuvant tamoxifen longer treatment might be better. In this respect the Scottish trial has shown significant survival benefit using a 5 year schedule and without late or enhanced toxicity. This trial has a secondary randomisation to continuing tamoxifen after 5 years or not and new trials are also addressing this question.

One of the most intriguing features of the NATO trial is the finding that survival benefit appears to be independent of oestrogen receptor status. This unexpected observation is not what would have been predicted from experience in advanced disease, and yet the Scottish trial reports a similar finding, at least in terms of disease-free survival. The issue is not entirely clear cut; for example the Danish trial found relapse-free survival benefit only in patients whose tumours had a high oestrogen receptor content ($>100\,\mathrm{fmol\,mg^{-1}}$) (Rose *et al.*, 1985). At the very least this remains a fascinating open question, and Michael Baum and his colleagues are to be congratulated for resisting the temptation to prejudge the issue; the premature decision of many other tamoxifen trials to exclude receptor negative patients has proved to be an important error.

At the end of the day histological grade rather than receptor status may end up as one of the main criteria for treatment selection. The paper in this issue from the NATO group on histological grade (p. 612) suggests quite strongly that the one group of patients who failed to benefit from tamoxifen were those with histological grade III tumours, despite the authors' claim that benefit here was simply 'quantitatively smaller' than for other grades. The current tendency to select adjuvant therapies simply on the basis of age or menopausal status seems biologically crude and a lot more data are needed on the relevance of grade, perhaps in association with EGF receptors (Sainsbury *et al.*, 1987) and other immunohistochemical markers, for treatment selection.

Finally, the NATO trial contains the germ of an idea that could carry the importance of tamoxifen beyond the boundaries of breast cancer. The number of deaths *not* due to breast cancer is less in the tamoxifen than in the control group (40 *versus* 61), and even with such small numbers this difference is significant. Indeed, the percentage reduction is greater than for breast cancer deaths (33% *versus* 16%). This could of course be a spurious chance finding, but it is intriguing that the Scottish trial comments without detail on a similar trend for non-cancer deaths. Tamoxifen is a complex drug that has oestrogenic as well as anti-oestrogenic effects. It is possible that these observations are pointing to a beneficial effect against osteoporosis, cardiovascular disease or some other as yet unidentified scourge of postmenopausal life. As time goes on the adjuvant tamoxifen trials may still have a lot to reveal.

**References**

BONADONNA, G., VALAGUSSA, B.S., ROSSI, A. & 4 others (1985). Ten-year experience with CMF-based adjuvant chemotherapy in resectable breast cancer. *Breast Cancer Res. Treat.*, **5**, 95.

BREAST CANCER TRIALS COMMITTEE (1987), Scottish Cancer Trials Office (MRC), Edinburgh. Adjuvant tamoxifen in the management of operable breast cancer: the Scottish Trial. *Lancet*, **ii**, 171.

BRINCKER, H., ROSE, C., RANK, F. & 4 others (1987). Evidence of a castration-mediated effect of adjuvant cytotoxic chemotherapy in premenopausal breast cancer. *J. Clin. Oncol.*, **5**, 1771.

JORDAN, V.C., MIRECKI, D.M. & GOTTARDIS, M.M. (1984). Continuous tamoxifen treatment prevents the appearance of mammary tumours in a model of adjuvant therapy. In *Adjuvant Therapy of Cancer*, Vol. 4, Jones, S.E. & Salmon, S.E. (eds) p. 27. Grune & Stratton: New York.

PADMANABHAN, N., HOWELL, A. & RUBENS, R.D. (1986). Mechanism of action of adjuvant chemotherapy in early breast cancer. *Lancet*, **ii**, 411.

ROSE, C., ANDERSEN, K.W., MOURIDSEN, H.T. & 4 others (1988). Beneficial effect of adjuvant tamoxifen in primary breast cancer patients with high oestrogen receptor values. *Lancet*, **i**, 16.

SAINSBURY, J.R.C., FARNDON, J.R., NEEDHAM, G.K., MALCOLM, A.J. & HARRIS, A.L. (1987). Epidermal-growth-factor receptor status as predictor of early recurrence of and death from breast cancer. *Lancet*, **ii**, 1398.